# Carcinoma of Testicle1Read at the thirtieth annual meeting of the Medical Association of Central New York, at Buffalo, October 19, 1897.

**Published:** 1898-08

**Authors:** A. A. Young

**Affiliations:** Newark, N. Y. 22 East Miller St.


### CARCINOMA OF TESTICLE.[1]

BY A. A. YOUNG, M. D., Newark, N. Y.

I DESIRE to state at the outset that the title of this paper is to be considered from the standpoint of the laity and thus including several malignant growths; this is intentional because the growth in question has not as yet been determined or classified. It is with this conception that I desire to report a case that came under my observation in March, 1897. As to the malignancy of the growth there can be no question, though no history of cancer or other hereditary disease could be elicited. The patient who had already passed seventy summers had always enjoyed moderately good health and during that period had been an active hard working man. The primary or exciting cause of this affection is therefore questionable and only conditions can therefore be considered.

Examination of the patient revealed an enlarged left testicle, fully four times as large as its fellow, hard, nodulated, tender and adherent on its anterior portion to the scrotum.

The adherent portion of the scrotum was circular in form and fully one and one half inches in diameter. An operation was at once advised and about ten days later was submitted to. The entire half of the scrotum was removed, including the septum scroti, which was also adherent to testicle. The amputation of the cord and accompanying vessels was made just within the inguinal canal. A suture or thread was passed through the cord, just above point of amputation and gentle, but continuous traction made upon the ligature in a downward direction, the object of which was to prevent the formation of a hernia, toward which there were marked tendencies. All bleeding vessels were secured and wound closed by sutures, save a small opening through which the ligature passed and this was plugged with iodoform gauze; repair was moderately rapid and satisfactory, save through that portion of tissue occupied by this suture. This suture was removed one week after the operation. For three weeks there seemed to be no improvement in the sinus, although it was freely irrigated with various antiseptic solutions. About the beginning of the fourth week application was made along the entire course of the sinus, by the aid of a bit of absorbent cotton on a probe, saturated

---

1 Read at the thirtieth annual meeting of the Medical Association of Central New York, at Buffalo, October 19, 1897.

with a 25 per cent. solution of formaldehyde (Merck). Repair at once began and it required only three applications, one every forty-eight hours, to practically close the sinus. Repair was soon completed and the parts assumed a healthful appearance and so remain today.

Of the medicinal agents protonuclein and red sulphuret of arsenic, 1-40 grain, were prescribed and persisted in for about two months, during which time no apparent benefit could be observed when they were discontinued.

About twenty weeks later the patient again appeared and infomed me that the remaining testicle was affected. It was about double its normal size, tender, but not nodulated or adherent to contiguous parts; by request of the patient, surgical interference was postponed for ten days, during which time the part materially increased in size and tenderness. The testicle was then removed, the cord severed just within the inguinal canal as before. The adjacent tissues not being involved were not sacrificed. The wound was closed and parts cleansed with a weak solution of formaldehyde daily. So far as the parts were concerned recovery was rapid and satisfactory. This wound differed from the other in that there was no pus formation.

The patient, however, from some cause rallied more slowly than from the previous operation. Anorexia and muscular weakness were marked. Several of the so-called tonics were tried without any apparent improvement in his condition until the administration of nuclein (Aulde) when happy and marked changes were observed, consisting of increased desire for food, together with its better assimilation and the consequent improvement in flesh and strength. The parts at present have the appearance of normal and healthful tissue. That the growth belongs to the class of encephaloid carcinoma is quite probable for we had the peculiar pain, rapid growth of the abnormal tissue, and its being confined to the body of the testicle and its becoming rapidly adherent to the scrotum. The left testicle, when first seen, was irregular in outline, due to the breaking down of the tunica albuginia; while the member seen last, where there was a short period of development, was swollen, but otherwise smooth and uniform.

There can be but little doubt but that my first impressions approximated the truth. As to treatment but little can be said outside of the time-honored custom of castration, which sometimes also proves a failure. As an adjunct to the "extirpating" treatment, from experience in this case, nuclein and formalin give flattering promises and each seem worthy of a more extended experimentation

22 East Miller St.

